# Cell-Specific DNA Methylation Patterns of Retina-Specific Genes

**DOI:** 10.1371/journal.pone.0032602

**Published:** 2012-03-05

**Authors:** Shannath L. Merbs, Miriam A. Khan, Laszlo Hackler, Verity F. Oliver, Jun Wan, Jiang Qian, Donald J. Zack

**Affiliations:** 1 Department of Ophthalmology, Johns Hopkins University School of Medicine, Baltimore, Maryland, United States of America; 2 Department of Molecular Biology and Genetics, Department of Neuroscience, and Institute of Genetic Medicine, Johns Hopkins University School of Medicine, Baltimore, Maryland, United States of America; 3 Institut de la Vision, Université Pierre et Marie Curie, Paris, France; University of Florida, United States of America

## Abstract

Many studies have demonstrated that epigenetic mechanisms are important in the regulation of gene expression during embryogenesis, gametogenesis, and other forms of tissue-specific gene regulation. We sought to explore the possible role of epigenetics, specifically DNA methylation, in the establishment and maintenance of cell type-restricted gene expression in the retina. To assess the relationship between DNA methylation status and expression level of retinal genes, bisulfite sequence analysis of the 1000 bp region around the transcription start sites (TSS) of representative rod and cone photoreceptor-specific genes and gene expression analysis were performed in the WERI and Y79 human retinoblastoma cell lines. Next, the homologous genes in mouse were bisulfite sequenced in the retina and in non-expressing tissues. Finally, bisulfite sequencing was performed on isolated photoreceptor and non-photoreceptor retinal cells isolated by laser capture microdissection. Differential methylation of *rhodopsin* (*RHO*), *retinal binding protein 3* (*RBP3, IRBP*) *cone opsin, short-wave-sensitive* (*OPN1SW*), *cone opsin, middle-wave-sensitive* (*OPN1MW*), and *cone opsin, long-wave-sensitive* (*OPN1LW*) was found in the retinoblastoma cell lines that inversely correlated with gene expression levels. Similarly, we found tissue-specific hypomethylation of the promoter region of *Rho* and *Rbp3* in mouse retina as compared to non-expressing tissues, and also observed hypomethylation of retinal-expressed microRNAs. The *Rho* and *Rbp3* promoter regions were unmethylated in expressing photoreceptor cells and methylated in non-expressing, non-photoreceptor cells from the inner nuclear layer. A third regional hypomethylation pattern of photoreceptor-specific genes was seen in a subpopulation of non-expressing photoreceptors (*Rho* in cones from the Nrl −/− mouse and *Opn1sw* in rods). These results demonstrate that a number of photoreceptor-specific genes have cell-specific differential DNA methylation that correlates inversely with their expression level. Furthermore, these cell-specific patterns suggest that DNA methylation may play an important role in modulating photoreceptor gene expression in the developing mammalian retina.

## Introduction

The vertebrate retina develops from an apparently homogeneous pool of pluripotent retinal neuroblasts [1]. Subsequent cellular differentiation is dependent upon precisely timed expression of cell type-specific genes under the synchronized and combinatorial influence of signaling molecules and transcription factors. To date, understanding gene regulation in the retina and the effects of intrinsic and extrinsic signaling molecules has primarily concentrated upon the role of specific DNA regulatory elements and the transcription factors with which they interact [2]. Epigenetic modifications, which can be persistent and heritable without directly changing the primary DNA sequence, provide an additional dimension to transcriptional regulation. Many studies have demonstrated that epigenetic mechanisms are important in the regulation of gene expression during embryogenesis, gametogenesis, and other forms of tissue-specific gene regulation [3]; however, little is known about the role of epigenetics in the establishment and maintenance of cell type-restricted gene expression in the retina [4].

One form of epigenetic regulation is DNA methylation. In vertebrates, methylation of the fifth carbon position of the cytosine residue in a 5′-CpG-3′ dinucleotide (CpG) is associated with a repressed chromatin state and inhibited gene expression [5]. About 70% of CpGs within the genome are methylated, and most unmethylated CpGs are found in CpG-rich sequences referred to as CpG islands [6]. CpG islands are primarily found in the 5′ regulatory region of genes, encompassing all or part of the promoter and can extend into or even beyond the first exon [7]. In general, ubiquitously expressed genes have unmethylated CpG islands close to 5′ of the TSS, and these regions remain unmethylated in all tissues and developmental states. Other regions of the genome show tissue-specific differential methylation, although the significance of this variance, especially for CpG-poor promoters, which are more often found in tissue-specific genes, remains unresolved [8]. These tissue-specific differentially methylated regions (T-DMRs), which have been identified by both restriction landmark genomic scanning [9]–[11] and microarray-based approaches [12]–[14], generally show an inverse relationship with differential gene expression. To investigate whether DNA methylation might play a role in the tissue-specific expression of photoreceptor genes, we investigated their methylation status in different tissues and cell types.

## Results

### DNA Methylation is Inversely Correlated with the Differential Expression of Retina-Specific Genes in Cultured Cell Lines

**Figure 1:**
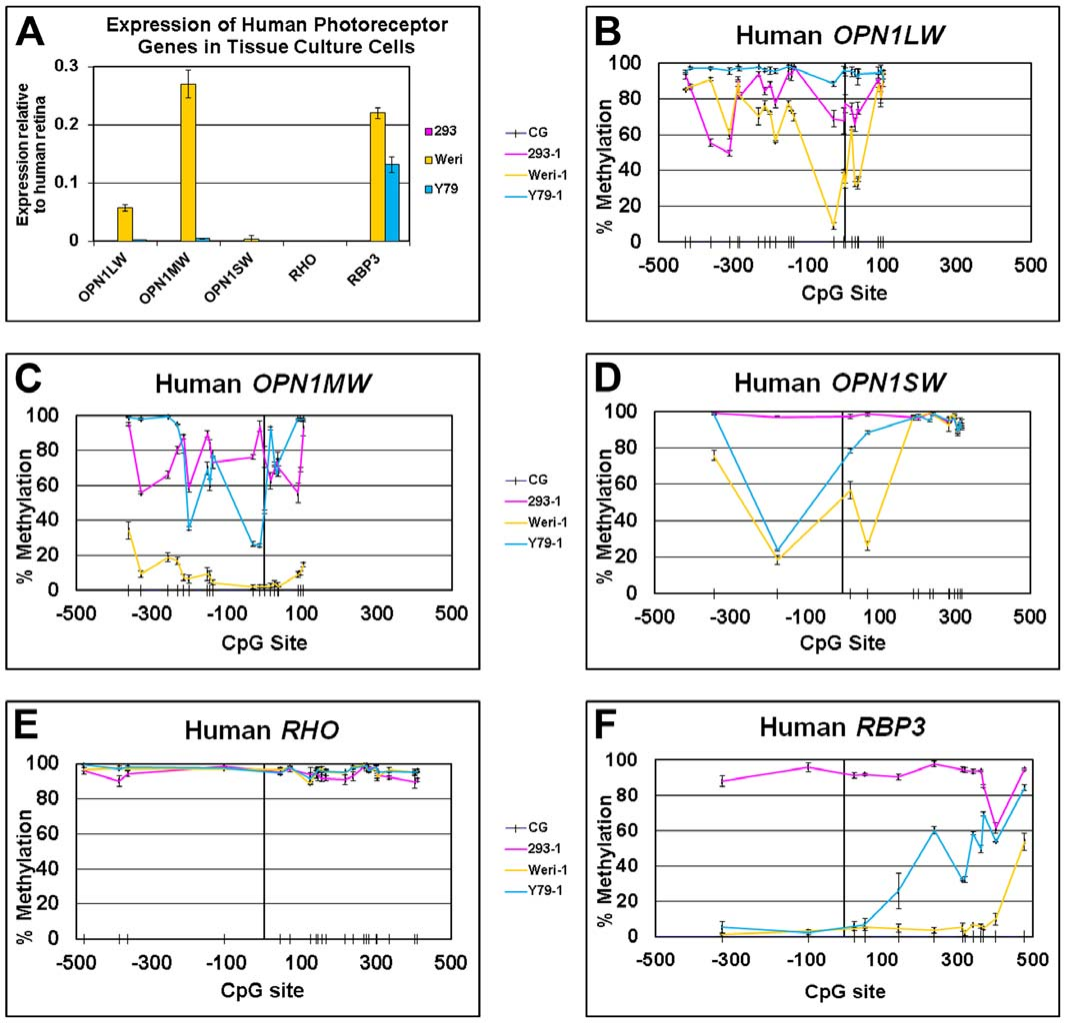
DNA methylation of photoreceptor genes in human cell lines is inversely correlated with expression levels. *A,* QPCR of *OPN1LW*, *OPN1MW*, *OPN1SW*, *RHO* and *RBP3* in Y79, WERI, and HEK293 cells, normalized to expression of human retinal cDNA. *B–F,* % DNA methylation of photoreceptor genes in 3 cell lines plotted versus the CpG site position (noted by hash marks) relative to the TSS (position “0”). Expression level and % methylation are the average of biological triplicates ± SEM.

We first sought to confirm and expand upon prior studies of the photoreceptor-specific gene expression in various cell lines. QPCR was performed for *RBP3* (Ensembl: ENSG00000107618), *RHO* (ENSG00000163914), *OPN1LW* (ENSG00000102076), *OPN1MW* (ENSG00000147380), and *OPN1SW* (ENSG00000128617) on the two human retinoblastoma (Rb) cell lines, WERI and Y79, and the human embryonic kidney cell line HEK293, which is known to have some neuronal features [15] (Fig. 1*A*). Both WERI and Y79 cells expressed *RBP3* [16], and WERI cells expressed the *OPN1LW* and *OPN1MW* at relatively high levels, consistent with previous reports [17]–[19]. Although clearly detectable and verified by their DNA melting curves, the expression of *OPN1MW* in Y79 cells and OPN1SW in WERI cells were both <0.5% of the expression levels seen in retina. Although *RHO* expression had been previously reported in Y79 cells [20], under our cell culture conditions, the expression of *OPN1LW*, *OPN1SW*, and *RHO* was undetectable. No detectable product in HEK293 cells was found for any of the photoreceptor genes.

We next wanted to determine if there was a relationship between these observed differences in gene expression and DNA methylation status. For this purpose we utilized bisulfite sequencing, in which sodium bisulfite modification of DNA is used to create sequence differences between methylated and unmethylated DNA by converting unmethylated cytosines to uracil, leaving methylated cytosines unchanged [21]. Partially methylated cytosines are proportionately represented by the C and T sequencing chromatogram peak heights after amplification. In the Y79, WERI, and HEK293 cells, bisulfite sequencing of the 500 bp upstream of the TSS through the end of the first exon for the photoreceptor genes *RHO*, *OPN1LW*, *OPN1MW* and *OPN1SW*, and 500 bp into the first exon for *RBP3*, demonstrated that the DNA methylation level of each gene (Fig. 1*B–F*) was inversely correlated with its level of expression. For *RBP3*, expressed in high levels in both WERI and Y79 cells, we observed a low level of methylation in WERI cells (Fig. 1*F*) for all the CpG sites between −500 bp and +500 bp except for site +478, which was approximately 50% methylated. A similar degree of methylation was seen for *RBP3* in Y79 for CpG sites −323 to +55, while sites +144 to +478 were approximately 50% methylated. In contrast, *RHO*, which was not expressed in any of the 3 cell lines, was densely methylated in all cases (Fig. 1*E*)). *OPN1SW* was minimally expressed in WERI cells, and CpG sites −348 to +59 were less methylated in WERI cells than in Y79 and HEK293 cells (Fig.1*D*). The region from 175 bp upstream of the *OPN1LW* TSS to the end of the first exon is homologous to the corresponding *OPN1MW* region. Although only 12 bp differ between the two genes, specific amplification of each gene was achieved for subsequent bisulfite sequencing. *OPN1MW*, expressed in WERI cells, minimally expressed in Y79 cells and not expressed in HEK293 cells, was relatively unmethylated in WERI cells (Fig. 1*C*). Only CpG sites −200, −30, −12 and +1 were more than 50% unmethylated in Y79 cells, and all CpG sites were densely methylated in HEK293 cells. *OPN1LW* was expressed exclusively in WERI cells, and CpG sites −231 through +28 were hypomethylated in WERI cells compared to Y79 and HEK293 cells (Fig. 1*B*). CpG sites −30 through +28 were less than 50% methylated, with the exception of CpG +19, which was relatively methylated (65%). Silent genes (all tested photoreceptor genes in HEK293, *RHO* in WERI, and *OPN1LW*, *OPN1SW*, *RHO* in Y79) were relatively methylated.

### Photoreceptor-Specific Genes are Hypomethylated in the Mouse Retina Relative to Other Tissues

**Figure 2:**
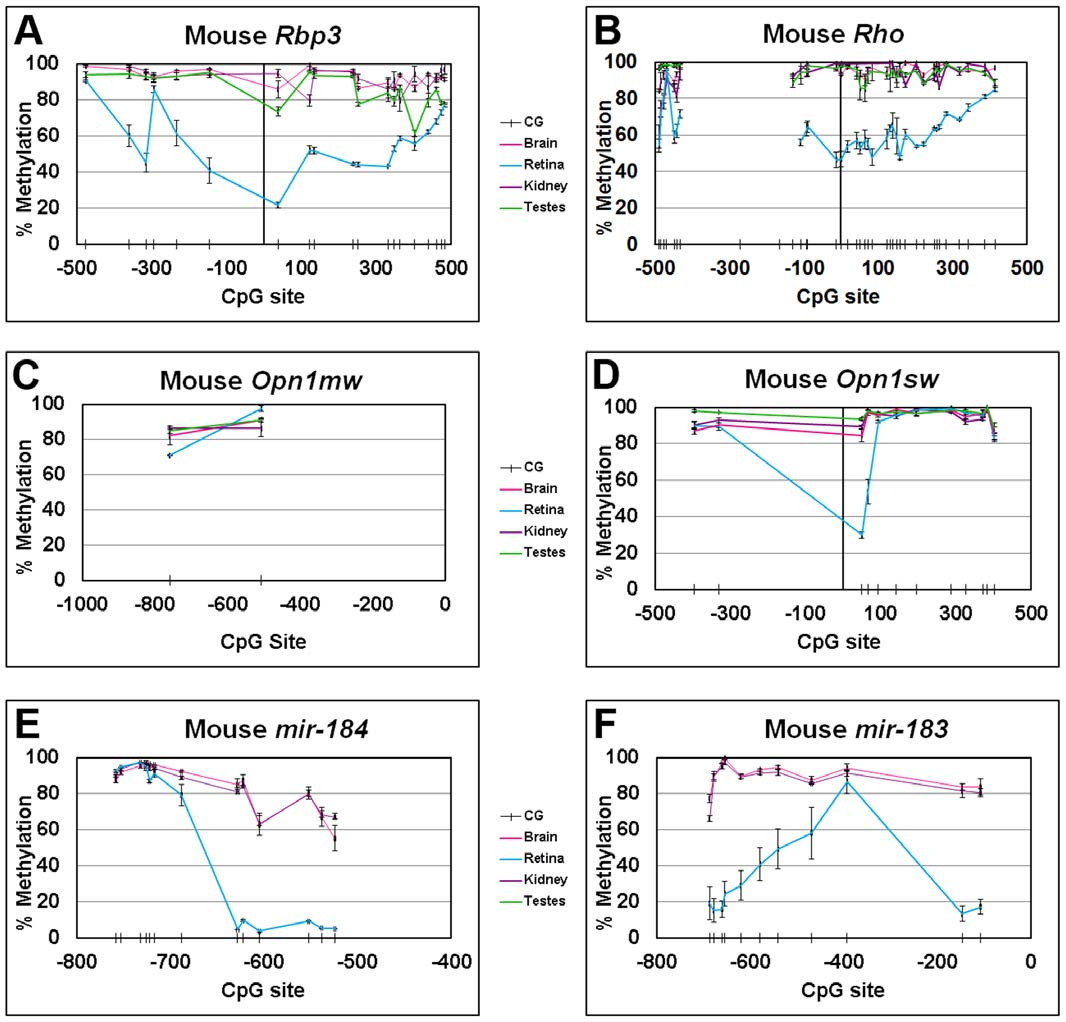
Bisulfite sequencing of mouse photoreceptor-specific genes and miRNAs shows increased DNA methylation in non-expressing tissues. *A–F,* Genomic DNA was isolated from 4 mouse tissues and bisulfite sequenced. % DNA methylation of photoreceptor genes was plotted versus the CpG site position (noted by hash marks) relative to the TSS (position “0”) for genes or the 5′ end of the miRNA stem-loop sequence (position “0”). % methylation is the average of 3 animals ± SEM. Reliable sequence was not obtained for *Rho* sites −270, −166, and −129 due to lack of sequence complexity that preceded these sites.

To extend the above cell culture-based observations of differential DNA methylation to the *in vivo* situation, the DNA methylation of orthologous mouse photoreceptor genes was examined in several representative mouse tissues. The majority of CpG sites around the TSS of both *Rbp3* (ENSMUSG00000041534) and *Rho* (ENSMUSG00000030324) were hypomethylated in the retina (a greater log_10_ (U/M)) relative to brain, kidney, and testes (Fig. 2*A–D*). The situation for the exclusively cone-expressed genes was different. For *Opn1sw* (ENSMUSG00000058831), only the two CpG sites closest to the TSS showed relative hypomethylation in the retina compared to other tissues (Fig. 2*D*). In the case of *Opn1mw* (ENSMUSG00000031394), there are no CpG sites within 500 bp upstream of the TSS or in the first exon, and the two CpG sites between 500 and 1000 bp upstream of the TSS were both methylated in all four tissues, including the retina (Fig. 2*C*). The % methylation observed in the mouse retina for *Rbp3* and *Rho* was higher than that observed for the orthologous genes in the human Rb cell lines (Fig. 2*A*–*B*), possibly reflecting differences between the *in vitro* and *in vivo* models and/or species-specific variation. Alternatively, since whole retina is comprised of approximately half photoreceptors (rods) which express *Rho* and *Rbp3* and half non-photoreceptors that do not, the 50% methylated state of most *Rho* and *Rbp3* CpG sites observed in retina may represent the average of two roughly equal cell populations - one unmethylated and one methylated. This possibility was explored by separately analyzing photoreceptors and non-photoreceptors isolated by laser capture microdissection (LCM) (see below).

### Putative Promoter Regions of miRNA Genes are Differentially Methylated in the Retina, Brain, and Kidney

Since there is increasing evidence that small regulatory RNAs such as miRNAs play a role in regulating retinal gene expression [22]–[25], as wells as data indicating that expression of miRNAs themselves is under epigenetic control [26], we explored the possible role of DNA methylation in regulating retinal miRNA expression. We determined the methylation status of the region upstream from the 5′ end of the stem-loop sequences of two miRNAs: *Mir183* (ENSMUSG00000065619) and *Mir184* (ENSMUSG00000065596). Both miRNAs are expressed in the retina but not in brain or kidney [27]. In both cases, we found clusters of CpG sites that were hypomethylated in the retina compared to the non-expressing brain and kidney (Fig. 2*E*–*F*).

### Retinal Neurons Demonstrate Cell-Specific Patterns of DNA Methylation

**Figure 3:**
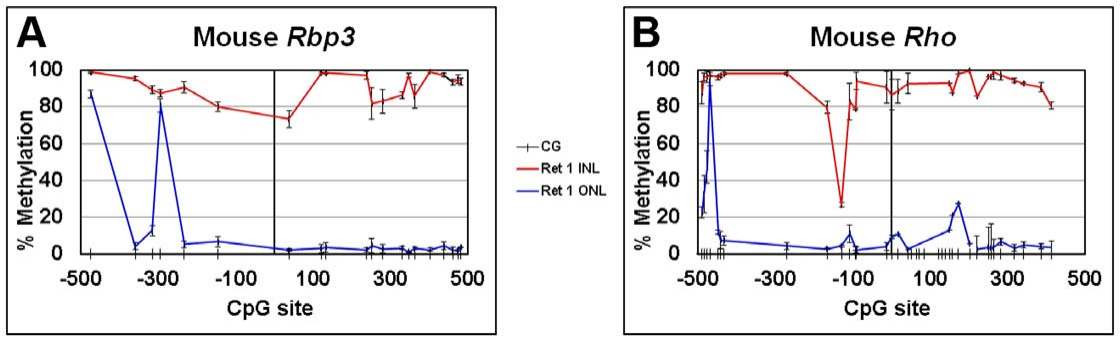
Photoreceptor-specific genes are hypomethylated in photoreceptors and methylated in non-photoreceptor cells from the INL. Cells from the ONL (expressing cells) and INL (non-expressing cells) were isolated by LCM from adult mouse retina. Genomic DNA was isolated and bisulfite sequenced. % DNA methylation of photoreceptor genes was plotted versus the CpG site position (noted by hash marks) relative to the TSS (position “0”) for *A*, *Rbp3* and *B*, *Rho*. % methylation is the average of 3 animals ± SEM.

To explore the methylation profile of *Rbp3* and *Rho* in the mouse retina in more detail, expressing photoreceptors from the ONL and non-expressing, non-photoreceptor cells from the INL were isolated by LCM from three different adult mouse retinas. Bisulfite sequencing of the genomic DNA from each cell population demonstrated that the *Rbp3* and *Rho* were mostly unmethylated around the TSS in photoreceptors (primarily rods) and mostly methylated in non-photoreceptor cells isolated from the INL (Fig. 3*A–B*), again demonstrating an inverse correlation between DNA methylation and cell-specific gene expression; however, not all *Rbp3* and *Rho* CpG sites sequenced were unmethylated in photoreceptors. CpG sites −475 and −294 in *Rbp3* and site −468 in *Rho* were methylated in photoreceptors, in addition to cells from the INL. In *Rho*, CpG site −128 had similar levels of methylation (∼20%) in both cell populations.

### Bisulfite Sequencing of Subcloned Fragments Reveals Correlation Between CpG Site Methylation

**Figure 4:**
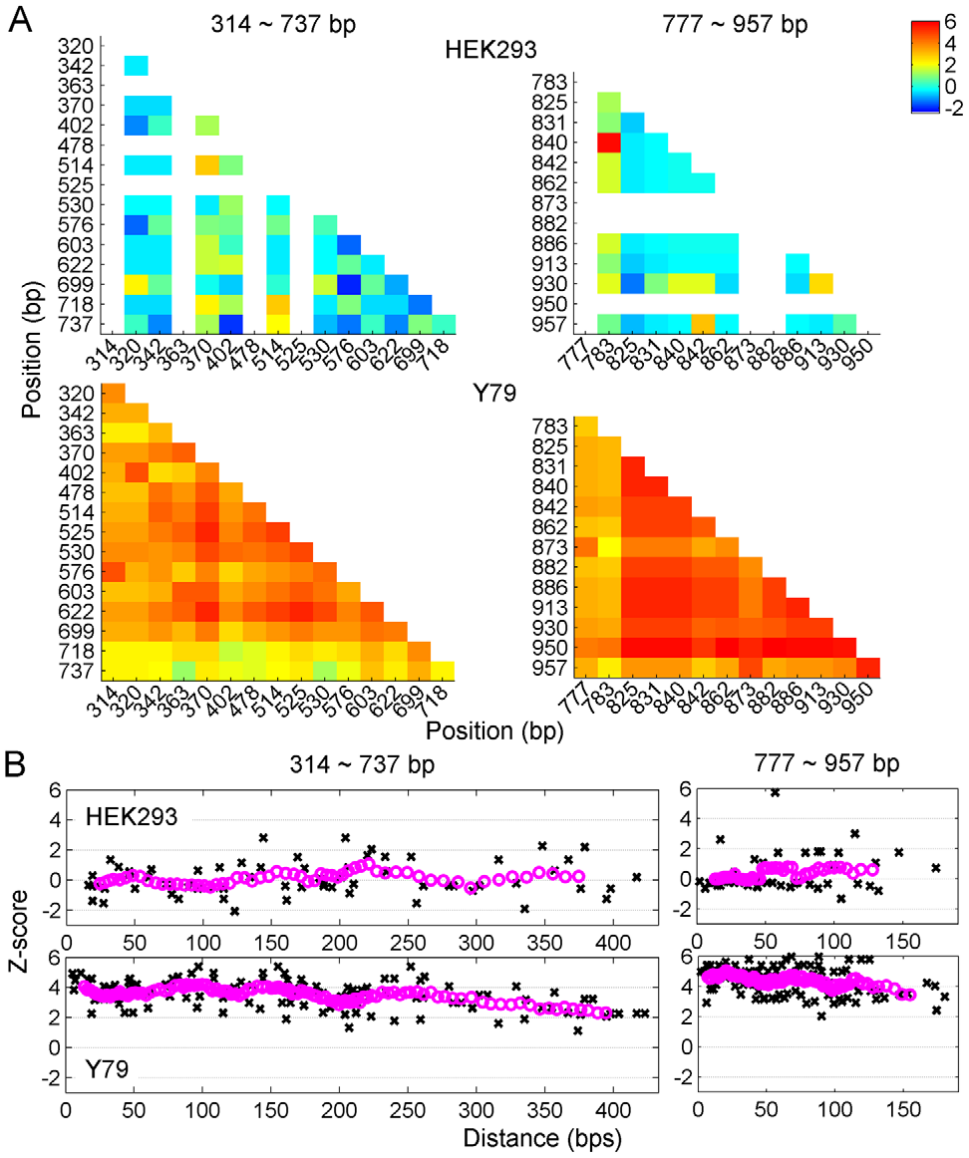
Z-score calculations show a variable correlation between the methylation status of RBP3 gene CpG sites. Z-score of correlation between methylated sites for *RPB3* in human cell lines HEK293 and Y79. *A*, Z-score of CpG site pairs (*RPB3* CpG sites 314–737, and 777–957 bp downstream of the TSS, which were amplified as 2 separate PCR products). There was no Z-score if one site was extremely hypermethylated or hypomethylated. Z-scores ranged from 6 (red) to −2 (dark blue). *B*, Z-score as function of distance between CpG site pairs in base pairs (bp). Magenta circles represent smoothed values within 9-point smoothing window.

**Figure 5:**
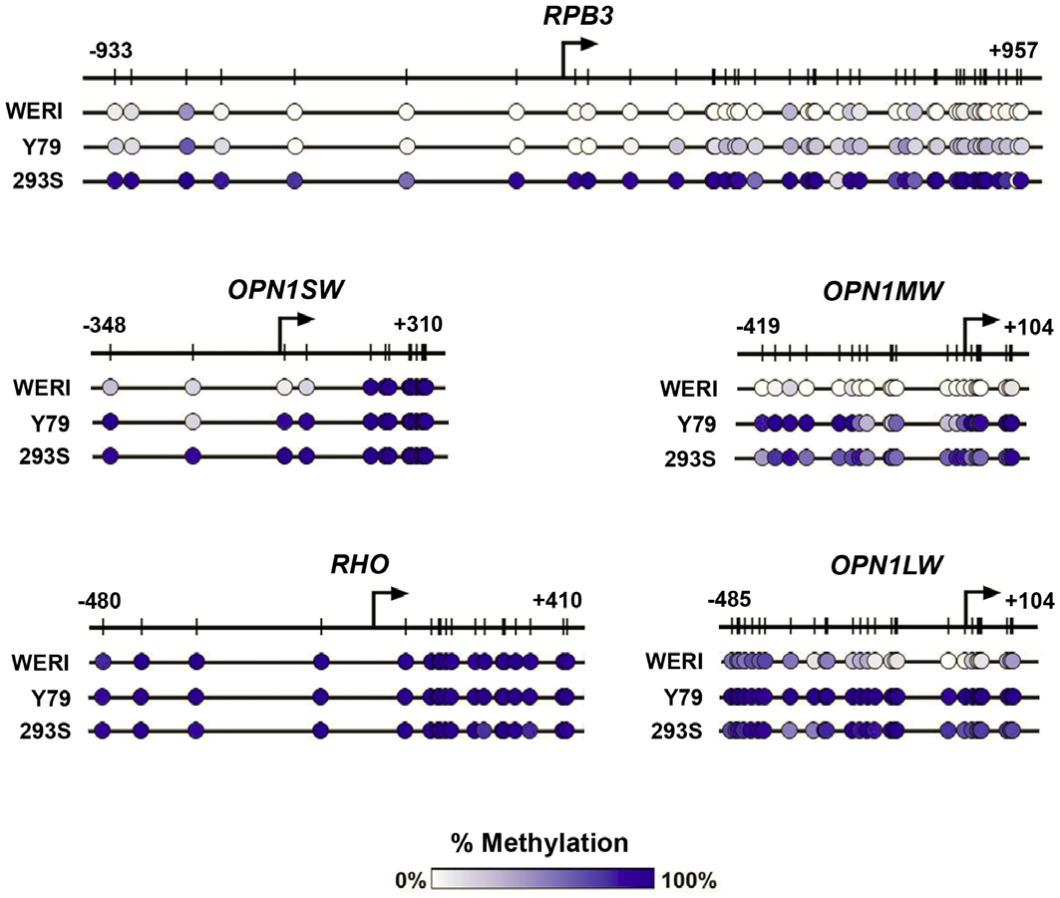
DNA hypomethylation correlates with expression of human photoreceptor-specific genes in tissue culture cells. Bisulfite sequencing of DNA samples from 3 different human tissue culture cell lines was performed. Thirty sequences (10 clones for each of 3 biological replicates) were batched together in a true-to-scale map, which represents the % methylation for each CpG site by a color gradient (white, unmethylated; dark blue, 100% methylated).

Direct sequencing of PCR amplified products from bisulfite-modified genomic DNA provides the average methylation at each CpG site for multiple DNA copies. This averaging masks specific DNA methylation patterns across multiple CpGs in a single DNA molecule, which could be particularly important for samples that are not completely methylated or unmethylated. To analyze molecule-specific DNA methylation patterns, the amplified fragment was subcloned and then individual clones were sequenced. By sequencing individual clones, correlation between separate intramolecular methylation sites could be identified. Rather than calculating the direct correlation between CpG sites, where if a pair of sites was extremely hypermethylated or hypomethylated, the correlation would be close to 1 but would not be statistically significant, the Z-score of correlation was calculated (see Methods S1). For *RBP3*, which had the greatest variation in DNA methylation of the human genes studied, some sites were identified as correlated, while other sites showed anti-correlation (Fig. 4*A*), indicating that DNA methylation of individual sites is not a random process, but rather that the methylation status of one site can influence that of another, positively in some cases, and negatively in others. The Z-score of correlation was plotted as a function of the distance between sites, normalized by expected correlation and variation for different methylation rates (Fig. 4*B*). Given the high degree and uniformity of the lack of methylation in WERI cells, a sufficient number of Z-scores could not be calculated for comparison. In HEK293 cells that did not express *RBP3*, there was no relationship between the Z-score and the distance between CpG sites. In contrast, an inverse correlation between the Z-score and the distance between CpG site pairs was identified in the expressing Y79 cells. Additionally, the bisulfite sequencing of subcloned fragments in the human tissue culture cell lines confirmed the direct sequencing results and showed that a lower % of DNA methylation correlated with gene expression (Fig. 5).

### Rod- and Cone-specific Genes Show Distinct DNA Methylation Patterns in Rod versus Cone Photoreceptor Cells

**Figure 6:**
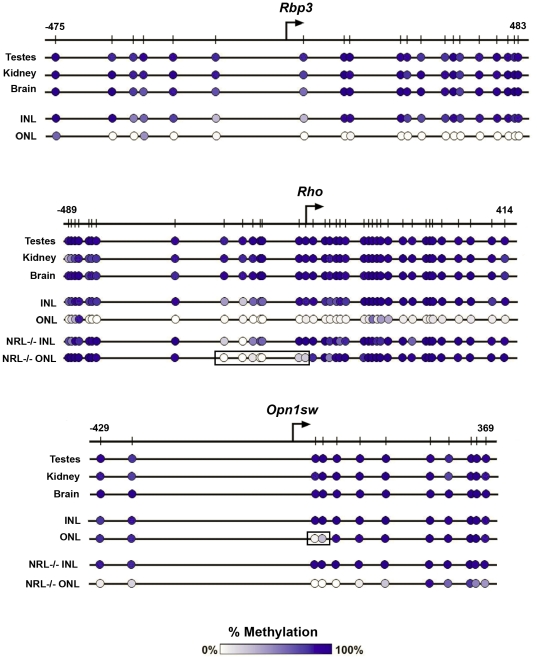
Hypomethylation of mouse photoreceptor-specific genes in expressing photoreceptors and regional hypomethylation in non-expressing photoreceptors. Bisulfite sequencing of photoreceptor-specific genes from non-expressing tissues (testes, kidney, and brain) and non-expressing cells from the INL, as well as expressing cells from the ONL (primarily rods from wild type mice and cones from Nrl −/− mice) was performed. Thirty sequences (10 clones for each of 3 biological replicates) were batched together in a true-to-scale map, which represents the % methylation for each CpG site by a color gradient (white, unmethylated; dark blue, 100% methylated). *Opn1mw* has only 2 CpG sites within the 1000 bp upstream of the TSS and none in the first exon. Both of these CpG sites (−762 and −510), were methylated in all tissues and cell types and the true-to-scale maps are not shown.

Bisulfite sequencing of photoreceptor-specific genes using the subcloning method, consistent with the PCR sequencing results, showed methylation in non-expressing tissues (testes, kidney, and brain) as well as non-expressing cells from the INL, but relative hypomethylation in the expressing cells from the ONL (Fig. 6). Specifically, the rod-expressed genes *Rbp3* and *Rho* were largely unmethylated in the ONL sample, which is primarily composed of rods in wild type mice. Likewise, the cone-expressed gene *Opn1sw* was unmethylated around the TSS in the ONL from Nrl −/− mice, which is composed primarily of cones as Nrl is required for rod photoreceptor development [28]. Interestingly, a third pattern of regional low level methylation distinguished rods from cones. In rods from the wild type ONL, there was regional hypomethylation near the *Opn1sw* TSS as compared to cells from the INL. Likewise, in cones from the Nrl −/− ONL, regional hypomethylation was observed just upstream of the *Rho* TSS as compared to cells from the INL. Similar, but less distinct hypomethylation was seen in the non-expressing cells from the INL for *Rho* and *Rbp3*. *Opn1mw* has only 2 CpG sites within the 1000 bp upstream of the TSS and no CpG sites in the first exon. Both of these CpG sites were methylated in all tissues and cell types.

## Discussion

Little is known about the function of DNA methylation and the role played by CpG islands in the establishment and regulation of tissue-specific expression. In other systems and tissues, tissue-specific differential methylation has been demonstrated by a variety of methods [9]–[14], and one possible role of these CpG sites within tissue-specific differentially methylated regions is the silencing by methylation of those genes that might oppose proper development of a particular tissue [29], [30]. Establishment of cell type-specific gene expression is an important milestone in retinal development. Here we have shown that vertebrate photoreceptor-specific and retinal-expressed miRNA genes demonstrate both cell-specific and site-specific DNA methylation patterns. Although the exact patterns differ in part because of the species differences in CpG location, the general hypomethylation pattern of expressed retinal genes is maintained between terminally differentiated retinal cells and immortalized cell lines. In the expressing cell lines, unmethylated CpG sites closest to the TSS (both up- and down-stream) correlate best with gene expression, while CpG sites further from the TSS remain methylated, suggesting that at least hypomethylation in this region is necessary for gene expression.

Despite the significant advances being made in understanding the role of epigenetics in gene regulation in other fields, little is known about the relationship between DNA methylation patterns and ocular development. Although the role that cell-specific DNA methylation plays in the retina is unclear, one possibility is that it helps direct proper lineage decisions and differentiation of retinal precursor cells. Recent evidence demonstrates that DNA methyltransferases (Dnmts) are involved in development of the vertebrate eye. High levels of mouse Dnmt expression are observed during early stages of retinal differentiation [31]. In the zebra fish embryo, knockdown of the maintenance Dnmt (*Dnmt1*) by a translation-blocking antisense morpholino results in a profound disorganization of all retinal layers [32]. Similarly, zebra fish mutants in *uhrf1 and dnmt1* have defects in lens development [33].

Previous studies using methyl-sensitive Southern blotting analysis of the promoter and first exon of *RBP3* demonstrated hypomethylation in expressing Y79 cells relative to human lymphocytes, which do not express *RBP3* [34]. Our studies confirm these findings and show that the hypomethylation extends both upstream and downstream from the TSS. These findings are also observed in other expressing cell lines, such as WERI, and at other expressed genes, like *OPN1MW*. Another study demonstrated that portions of the *Rbp3* promoter in the adult mouse are specifically unmethylated in the retina but methylated in non-expressing tissues [35]. We extended this observation to include all of the CpG sites 500 bp up- and down-stream of the TSS for *Rbp3*, as well as for 3 other photoreceptor-specific genes, *Rho*, *Opn1sw*, and *Opn1mw*. We found these genes to be relatively unmethylated in expressing cells and densely methylated in non-expressing cells, irrespective of whether they were part of a CpG island or located in a CpG-poor region. For example, *Rho* has a clearly defined 5′-CpG island, while *Rbp3* does not, and yet we found that both genes show photoreceptor-specific unmethylation around the TSS. Our results are also consistent with the low level of methylation of the *OPN1MW* and *OPN1LW* promoters previously seen in the WERI cells [36].

Now that we have defined some of the specific topology of DNA methylation in select photoreceptor-specific genes and miRNA genes in the adult retina, it will be important to determine how these patterns arise during retinal development in the mouse. Another interesting model in which to study how methylation patterns change is the differentiation *in vitro* of embryonic stem (ES) and induced pluripotent stem (iPS) cell-derived retinal precursors into more differentiated retinal cells. Whether DNA methylation plays a direct and causal role in retinal development by gene silencing or merely represents a “marker” for silent chromatin after expression is repressed through other mechanisms [37] is unclear, but such developmental studies may provide some insight. The observation that a lack of DNA methylation of the mouse *Rbp3* promoter occurs just before *Rbp3* gene activation in the developing retina [38], suggests that this demethylation of the promoter may be partially responsible for *Rbp3* gene activation and/or may modulate its expression level. One explanation for the observed DNA methylation differences between rods and cones (i.e. the regional methylation pattern observed near the TSS of *Opn1sw* in rods and *Rho* in cones) is that it represents the methylation signature of photoreceptor-specific precursor cells. If this were the case, once cell fate was determined, these early methylation patterns would become completely unmethylated in expressing photoreceptors but persist in the non-expressing photoreceptors. In addition to studies on the timing of differential methylation in the developing retina, use of technologies that allow the site-specific modulation of DNA methylation *in vivo* [39]–[43] will likely also be important in defining the mechanistic importance of DNA methylation in regulating and modulating linage-specific gene expression in the retina.

## Materials and Methods

### Ethics Statement

This study was specifically approved by the Johns Hopkins University Institutional Animal Care and Use Committee (IACUC), and the Johns Hopkins University IACUC approval number is MO 09M281.

### Cell Lines and Cell Culture

HEK293, Y79, and WERI cells were purchased from American Type Culture Collection (Manassas, VA). Y79 and WERI cells were maintained in RPMI 1640 supplemented with 10% fetal bovine serum (FBS), 10 mM Hepes, 2 mM L-glutamine, and 100 units/mL penicillin and streptomycin. HEK293 cells were maintained in Dulbecco's modified Eagle's medium supplemented with 10% FBS, 2 mM L-glutamine, and 100 units/mL penicillin and streptomycin. Media, supplements and antibiotics for tissue culture were purchased from Invitrogen (Carlsbad, CA).

### Real-time Quantitative PCR (QPCR)

Total RNA from the Y79, WERI, and HEK293 cell pellets was isolated with the RNeasy Mini kit (Qiagen, Valencia, CA). Total RNA from human retina obtained from the National Disease Research Interchange was isolated with TRIzol (Invitrogen, Carlsbad, CA). cDNA was synthesized in a 20 µL reaction using 500 ng total RNA and SuperScript III (Invitrogen, Carlsbad, CA) according to the manufacturer's instructions. Primers were designed with the Universal ProbeLibrary Assay Design Center (www.roche-applied-science.com) to span introns of *RPB3*, *RHO*, *OPN1SW*, *OPN1MW*, and *OPN1LW* (Table S1). A BioRad IQ5 Multicolor Real Time PCR Detection System was used for analysis, and relative expression was calculated with the Pfaffl method [44] normalized to GAPDH.

### Laser Capture Microdissection (LCM)

Eyes from 2 month old C57BL/6J mice or 1 month old Nrl −/− mice (a kind gift from Anand Swaroop, National Eye Institute) [45] were isolated and the lenses removed. The eye cups were cryoprotected by passage through increasing (6.75%–25%) concentrations of ice-cold sucrose in 0.1 M phosphate buffer, equilibrated with a 2∶1 mixture of 25% sucrose/OCT (Tissue Tek) for 1 h at 4°C, snap frozen on dry ice, and cryosectioned at −30°C. Seven µm sections were thaw-mounted onto PEN foil slides, kept at −30°C for 10–30 min, fixed in ice-cold 70% ethanol for 30 sec, rinsed in water, stained in Meyer's hematoxylin (2 min), dehydrated through a 70%–95%–100% ethanol series (30 sec each), and air dried for 2 min. Cells from the outer nuclear layer (ONL – photoreceptors) and the inner nuclear layer (INL – non-photoreceptor neurons) were separated by LCM using a Leica LMD6000 system. Cut tissue fragments were collected by gravity into tube caps containing lysis buffer (Qiagen kit) for DNA isolation.

### Genomic DNA Isolation

For all experiments, genomic DNA was isolated from biological triplicates. The DNeasy Tissue kit (Qiagen, Valencia, CA) was used for the cell line pellets (Y79, WERI, and HEK293) and the whole tissue samples (retina, brain, kidney and testes harvested from 3 different 2 mo old C57BL/6J mice). The DNA Micro kit (Qiagen, Valencia, CA) was used for the ONL and INL samples isolated by LCM from 3 different 2 mo old C57BL/6J mice and 3 different 1 mo old Nrl −/− mice.

### Bisulfite Sequencing

Bisulfite modification of genomic DNA was performed using either the Epitech (Qiagen, Valencia, CA) or EZ DNA Methylation (Zymo, Irvine, CA) kits. MethPrimer [46] was used to design primers to specifically amplify photoreceptor specific genes and the putative promoter regions of two intergenic miRNAs (*Mir183* and *Mir184*) (Tables S2, S3, S4). For direct sequencing, the PCR products were agarose gel-purified and directly sequence by the High-Throughput Genomics Unit at the University of Washington using the forward primers. Using the ContigExpress function of Vector NTI Advance 10 (Invitrogen, Carlsbad, CA), the chromatograph peak heights for C and T at each CpG site were measured and the % methylation (C peak height/(T peak height+C peak height)) versus CpG position, with respect to the TSS, was plotted [47]. For sequencing of individual clones after PCR amplification, the presence of a specific PCR product was confirmed by agarose gel electrophoresis, and the PCR product for each set of primers was subcloned using the TOPO-TA cloning kit (Invitrogen, Valencia, CA). Thirty sequences (10 clones for each of 3 biological replicates) with ≥95% conversion of the non-CpG cytosines were obtained and sequenced using the M13 reverse primer by the University of Washington High-throughput Genomics Unit. Fasta files of each sequence were analyzed using a custom JAVA program (DNAMethylMap, unpublished software by B. Kumar and S. Yegnasubramanian).

### Calculation of Methylation Correlation

A Z-score of methylation correlation was calculated (Methods S1). Larger positive Z-scores indicate that the methylated sites are more correlated than would be expected based upon random permutation analysis, whereas smaller negative Z-scores indicate less correlation than would be expected from the permutation analysis.

## Supporting Information

Table S1 **Human QPCR Primers.**
(DOC)

Table S2 **Human Bisulfite Sequencing Primers.**
(DOC)

Table S3 **Murine Bisulfite Sequencing Primers.**
(DOC)

Table S4 **Murine miRNA Bisulfite Sequencing Primers.**
(DOC)

Methods S1 (DOC)
