# Supplementary material for: Cell-Specific DNA Methylation Patterns of Retina-Specific Genes
Source: PLoS One. 2012 Mar 5;7(3):e32602. doi: 10.1371/journal.pone.0032602 (PMC3293830; doi:10.1371/journal.pone.0032602)
Supplement: Table S1 — Human QPCR Primers. (DOC) [file pone.0032602.s001.doc]

**Table S1. Human QPCR Primers**

| **Gene** | **Exon** | **Sequence (5' - 3')** |
| --- | --- | --- |
| ***OPN1SW*** | 3 | ATACCGCAGCGAGTCCTATAC |
|  | 4 | GATCCTACCATCACAACCAC |
| ***OPN1MW*** | 2 | CTACACCGTCTCCCTGTGTG |
|  | 3 | GCTTGCAGACCACCATCC |
| ***OPN1LW*** | 4 | CGCTATCATCATGCTCTGCT |
|  | 5 | AGACTCTTTCTGCTGCTTTGC |
| ***RHO*** | 4 | TCATCTATATCATGATGAACAAGCAG |
|  | 5 | GCCTCATCGTCACCCAGT |
| ***RBP3*** | 2/3 | CCATGATCATCGACATGAGG |
|  | 3 | TCAAAGAAGTAGGAGCACAAGATG |
| ***GAPDH*** | 6 | CTGACTTCAACAGCGACACC |
|  | 6/7 | TAGCCAAATTCGTTGTCATACC |
