# Supplementary material for: Cell-Specific DNA Methylation Patterns of Retina-Specific Genes
Source: PLoS One. 2012 Mar 5;7(3):e32602. doi: 10.1371/journal.pone.0032602 (PMC3293830; doi:10.1371/journal.pone.0032602)
Supplement: Table S2 — Human Bisulfite Sequencing Primers. (DOC) [file pone.0032602.s002.doc]

Table S2. Human Bisulfite Sequencing Primers

| **Gene** |  | **Oligo Name** | | **Start~** | | **Sequence (5' - 3')** | |
| --- | --- | --- | --- | --- | --- | --- | --- |
| ***OPN1SW*** |  | B F4 | | -475 | | TTT TTT TGT AGT TTA GGG TTG GTT TTT AG | |
|  |  | B R4 | | -13 | | CCC CCT CTA AAT CCT CTT ATA AAT AAT | |
|  |  | B F3 | | -150 | | TGG TTT AGG GAT TTT TAA TTT TAA ATT TTG | |
|  |  | B R2 | | 350 | | ACT ACA ATA CCC AAA AAA CCC TC | |
| ***OPN1MW*** |  | G F3 | | -478 | | GTT GGG AGT ATA GGT ATT TGT TAT TAA GTT | |
|  | ! | G R1 | -137 | | CC**C** CTT ACT **T**CC **T**TA CTC CTA AAA C | |  |
|  | **!** | G F4 | | -136 | | TTT TAG GAG TA**A** GG**A** AGT AAG **G** | |
|  | ***** | G R2 | | 130 | | AAA TAC TAT CCT CAT AAC TAT CCT AC | |
| ***OPN1LW*** |  | RED F3 | | -526 | | GTA GTT AGT TTG TTT TTT TTA TAT TGG AGG | |
|  | **!** | RED R3 | | -112 | | CC**T** CTT ACT **C**CC **C**TA CTC CTA AAA C | |
|  | **!** | RED F4 | | -136 | | TTT TAG GAG TA**G** GG**G** AGT AAG **A** | |
|  | ***** | G R2 | | 130 | | AAA TAC TAT CCT CAT AAC TAT CCT AC | |
| ***RHO*** |  | R F1a | | -635 | | TTT GAG TTT TTT TGG GTA GGG TTG | |
|  |  | R R1a | | -330 | | ACT TTC TAA TTT ATT CTC CCA ATC TCT C | |
|  |  | R F5a | | -147 | | GGT TTT TTT TAG AAG TTA ATT AGG TTT TTA G | |
|  |  | R R4 | | 436 | | ACC CTC CAA ATT ACA TCC TAT AAA C | |
| ***RBP3*** |  | I F1b | | -1037 | | TAG GAG TTG GGA TTT GAA GAG TTT A | |
|  |  | I R1b | | -647 | | AAC ATC AAC CTC AAA ACT TAA AAT TTA T | |
|  |  | I F5a | | -671 | | ATT TTA AGT TTT GAG GTT GAT GTT AG | |
|  |  | I R5 | | -299 | | AAA CTA TTC TAC CAC TAT CTC TAC C | |
|  |  | I F6a | | -240 | | AGT TTG TAA AGG ATG GAG TAT AGT GTT TG | |
|  |  | I R6b | | 302 | | CTC AAA ATC TCA TAA CTC TTA ATA ACC TAC | |
|  |  | I F7 | | 241 | | GAG AAT TTG TTG GGT ATG TAG GAA G | |
|  |  | I R7 | | 765 | | CAA AAC CTA AAA CAA AAT CCA AAT C | |
|  |  | I F4a | | 751 | | TTG TTT TAG GTT TTG GGA GAA AGG | |
|  |  | I R4a | | 997 | | TCT AAC TAC CTC CAC CAA AAA ACC | |

Oligo, oligonucleotide

~ Start position with respect to TSS

! Although *OPN1MW* and *OPN1LW* share are significantly homologous around the TSS, we were able to specifically amplify the homologous target region from each gene by incorporating the gene-specific SNPs highlighted in bold into the design of primers G R1 and Red R3, and Red F4 and G F4.

***** This primer binds non-specifically to both *OPN1MW* and *OPN1LW* within the region of homology but is paired with primers Red F4 or G F4 that do bind differentially to *OPN1LW* and *OPN1MW.*
