# Supplementary material for: Cell-Specific DNA Methylation Patterns of Retina-Specific Genes
Source: PLoS One. 2012 Mar 5;7(3):e32602. doi: 10.1371/journal.pone.0032602 (PMC3293830; doi:10.1371/journal.pone.0032602)
Supplement: Table S3 — Murine Bisulfite Sequencing Primers. (DOC) [file pone.0032602.s003.doc]

**Table S3. Murine Bisulfite Sequencing Primers**

| **Gene** | **Oligo Name** | **Start~** | **Sequence (5' - 3')** |
| --- | --- | --- | --- |
| ***Opn1sw*** | MB F1a | -67 | TGA GAA TTA AGA GAT TTT TAA TTT GGG |
|  | MB R1 | 427 | AAA ACC TCC AAA ACA CAA ACA TAA C |
|  | MB F2 | -526 | GAA GTA GTT TAA TTT GGT TGG TTG TAG |
|  | MB R2 | -296 | CAC CTA AAT ATC CCT ATC CAA AAA C |
| ***Opn1mw*** | MG F1 | -826 | TTG TTT GGA AGT GGT TAT GTT TA |
|  | MG R1 | -444 | AAC AAT TTA TTA ATA AAC CCC AAA AAT AC |
| ***Rho*** | MR F1Na | -205 | GAG ATA TTT TTT TTT TTT TTT ATT TAA GGG |
|  | MR R1N | 115 | AAA ACA CAT AAA AAT TAA AAC CCT CTA |
|  | MR F3 | 85 | GGT ATA GAG GGT TTT AAT TTT TAT GTG |
|  | MR R3 | 451 | CTC TAC TCA TAC CTC CAA ATA TAA C |
|  | MR F4 | -532 | TAT GTG AGT GAA TAA ATT TTA ATT GAA |
|  | MR R4 | -142 | CTT ACA AAA AAA CCA AAA TAA CAT C |
| ***Rbp3*** | MI F3 | 84 | AGA ATG GGT TTT GGT TTT GTT TAT AT |
|  | MI R3 | 536 | TAA CTC ACT AAA AAC TCC CCC |
|  | MI F4a | -347 | TTA GAG TTA TGG GTG AGT TAT AGT G |
|  | MI R4a | 183 | CAA AAA AAT CTT AAC CAT ATC CAA TAC |
|  | MI F1a | -543 | AAA TTT TTT GGT GAT AAA ATA GAT ATG ATA |
|  | MI R1a | -60 | AAC TTC CAA CTC TAC TAA ACC TTT AAT C |

Oligo, oligonucleotide

**~** Start position with respect to TSS
