# Supplementary material for: Cell-Specific DNA Methylation Patterns of Retina-Specific Genes
Source: PLoS One. 2012 Mar 5;7(3):e32602. doi: 10.1371/journal.pone.0032602 (PMC3293830; doi:10.1371/journal.pone.0032602)
Supplement: Table S4 — Murine miRNA Bisulfite Sequencing Primers. (DOC) [file pone.0032602.s004.doc]

**Table S4. Murine miRNA Bisulfite Sequencing Primers**

| **MiRNA name** | **Frag Name** | **Start~** | **Sequence (5' - 3')** |
| --- | --- | --- | --- |
| **miR184** | **184-12** | -667 | GTG TTA GTA GAA AGG ATT TGA TAA GAT |
|  |  | -474 | ACC AAA ATT AAT CCT CCA AAA AAA C |
|  | **184-13** | -800 | GTT GAG TTT TGG AAT AGT TTT TTT TG |
|  |  | -629 | TCT CAC TAT CTC ATC TTA TCA AAT CCT T |
| **miR183** | **183-9** | -713 | GTA AGG GTT TTT ATT TTG TTT TTT T |
|  |  | -368 | TCC AAT ATA CTT CTC ATA ACC TCT |
|  | **183-14** | -1960 | TTT TAA TTT TAA GGT TAG GGT TTT G |
|  |  | -1761 | CAA CAA AAC AAA AAT ACC TAA ACC |
|  | **183-15** | -1772 | TTT GTT TTG TTG TGT GTT GG |
|  |  | -1542 | CCC TTT TAC CTA CAT CTT CCT C |
|  | **183-16** | -1639 | GGA TTA GAA TTG GAT GTT GAA G |
|  |  | -1465 | CTC AAT CTA AAA CAC CCA AAA A |
|  | **183-17** | -1374 | GGG GAA AAA GAT TTT AGA ATA TTG |
|  |  | -1079 | CAC TCC CTA ACC TAT AAC CTC A |
|  | **183-18** | -1126 | GTT TAG AGA TTT ATA GTG GGG GAA |
|  |  | -777 | CCC AAA ACA TAA AAA CTA AAA ACA C |
|  | **183-19** | -251 | GAT GTG ATG GGA AAT TTT GTT |
|  |  | -7 | AAT CAC ACT CTC CAA CCT ACA A |

Frag, fragment

**~**Start position with respect to the 5` end of the stem-loop sequence
