# Supplementary material for: Cell-Specific DNA Methylation Patterns of Retina-Specific Genes
Source: PLoS One. 2012 Mar 5;7(3):e32602. doi: 10.1371/journal.pone.0032602 (PMC3293830; doi:10.1371/journal.pone.0032602)
Supplement: Methods S1 — (DOC) [file pone.0032602.s005.doc]

**Methods S1**

DNAMethylMap performs a BLAST alignment of the sequence data from each clone and sample to a virtually bisulfite converted reference sequence, assuming all CpGs, but no other cytosines, are methylated. These alignments are then used to output rates of bisulfite conversion of each sequence for quality control purposes, rates of CpG methylation, and true-to-scale maps of methylated and unmethylated CpGs represented by open and closed circles. Thirty sequences (10 clones for each of 3 SUPbiological replicates) from the same reference gene and tissue type with ≥95% conversion of the non-CpG cytosines were batched together in the output figure, which represents the % methylation for each CpG site by a color gradient.

Correlation of methylation between *i* and *j* was calculated such that

$$Cr_{i,j}=1-2\times\frac{\sum_{k=1}^{N} \left|M_{ik}-M_{jk}\right|}{N},$$

where *N* is total number of alleles, *M_ik_* = 1 (methylated) or 0 (unmethylated) for each allele *k* at site *i*. *Cr = 1* represents perfectly correlated CpG sites, whereas *Cr = -1* refers to anti-correlation. Given that *n_i_* and *n_j_* alleles were methylated at site *i* and *j*, respectively, and $x_{ij}$ of alleles among them were methylated at both sites

$$Cr_{ij}\left(x_{ij}\left|n_{i},n_{j}\right.\right)=1-2\times\frac{n_{i}+n_{j}-2x_{ij}}{N}.$$

If a pair of sites was extremely hypermethylated or hypomethylated, the correlation will be close to 1; however, the correlation is not statistically significant under these circumstances. To evaluate the deviation of observed correlation from expectation, the Z-score of correlation was calculated

$$Z=\frac{Cr_{ij}\left(x_{ij}\left|n_{i},n_{j}\right.\right)-\overline{Cr_{ij}\left(n_{i},n_{j}\right)}}{SD_{ij}\left(n_{i},n_{j}\right)},$$

where $SD_{ij}\left(n_{i},n_{j}\right)$ is the standard deviation of $Cr_{ij}\left(x_{ij}\left|n_{i},n_{j}\right.\right)$ for all $x_{ij}$, the expectation $\overline{Cr_{ij}\left(n_{i},n_{j}\right)}$ is the average correlation of all possible combinations for a given set given by

$$\overline{Cr_{ij}\left(n_{i},n_{j}\right)}=\sum_{x_{ij}} Cr_{ij}\left(x_{ij}\left|n_{i},n_{j}\right.\right)\times p\left(x_{ij}\left|n_{i},n_{j}\right.\right),$$

where $p\left(x_{ij}\left|n_{i},n_{j}\right.\right)$ can be calculated based on a hypergeometric model

$$p\left(x_{ij}\left|n_{i},n_{j}\right.\right)=\frac{\left(\begin{matrix} n_{i} \\ x_{ij} \end{matrix}\right)\left(\begin{matrix} N-n_{i} \\ n_{j}-x_{ij} \end{matrix}\right)}{\left(\begin{matrix} N \\ n_{j} \end{matrix}\right)},$$

which reflects the possibility of $x_{ij}$ clones methylated at both sites for a given set of .
